# Development of male and female models of long urethral strictures in swine

**DOI:** 10.1016/j.sopen.2023.11.002

**Published:** 2023-11-20

**Authors:** Gokhan Gundogdu, Travis Nguyen, Mando Eijansantos, Ambika Chaudhuri, David Barham, Joel Gelman, Joshua R. Mauney

**Affiliations:** a Department of Urology, University of California, Irvine, Orange, CA 92868, USA; b Department of Biomedical Engineering, University of California, Irvine, Irvine, CA 92617, USA

**Keywords:** Urethral stricture, Tissue engineering, Wound healing, Swine

## Abstract

**Background:**

Preclinical animal models which mimic the dimensions of long urethral strictures (>2 cm in length) encountered in the clinic are necessary to evaluate prospective graft designs for urethroplasty. The purpose of this study was to develop both male and female porcine models of long urethral strictures (∼4 cm in length) and characterize histological and functional outcomes of iatrogenic stricture formation between genders.

**Methods:**

Focal, partial thickness urethral injuries were created over 5–6 cm long segments in male and female swine (*N* = 4 per gender) via electrocoagulation and the degree of stricture formation was monitored for up to 6 weeks by urethroscopy and retrograde urethrography. Animals were sacrificed following stricture confirmation and histological, immunohistochemical, and histomorphometric analyses were performed on strictured and uninjured control urethral segments to profile wound healing responses.

**Results:**

Urethral stricture formation was detected in all female swine by 2 weeks and 100 % of male swine at 3.2 ± 1.8 weeks, post-operatively. The mean length of urethral strictures in both male and female swine was ∼4 cm. Substantial variations in the degree of stricture severity between sexes were observed with males exhibiting significant urethral stenosis and loss of α-smooth muscle actin+ smooth muscle bundles in comparison to controls, while females primarily displayed defects in pan-cytokeratin+ epithelia as well as functional urethral obstruction.

**Conclusions:**

Electrocoagulation injury is sufficient to produce long urethral strictures in male and female swine and the degree of stricture severity and nature of urethral obstruction was observed to be dependent on gender. Animal Protocol: AUP-19-150.

**Key message:**

Novel male and female models of long urethral strictures in swine were created to characterize histological and functional outcomes of iatrogenic stricture formation between genders.

## Introduction

Urethral stricture disease is a significant public health issue which occurs due to scarring in or around the urethra that restricts or blocks urine flow. Patients with urethral strictures are responsible for 5000 hospital and 1.5 million office visits per year in the United States and are considered a vulnerable population as they experience high rates of urinary tract infections (41 %) and incontinence (11 %) as sequelae of the disease [1,2]. Urethral stricture disease is also well documented to significantly reduce patient quality of life [3], particularly in men wherein up to 44 % of individuals experience sexual dysfunction [4]. Urethral strictures are relatively common in males (∼400 per 100,000) with increased incidence after 55 years of age [5]. Men with symptomatic stricture disease will typically present with obstructive voiding symptoms such as straining, incomplete emptying, weak stream, recurrent urinary tract infections, prostatitis, epididymitis, hematuria, or bladder stones [6]. Stricture etiologies in men include iatrogenic caused by prolonged catheterization and prior hypospadias repair; infection or inflammation due to sexually transmitted diseases or lichen sclerosis; trauma including straddle injuries or pelvic fractures; and congenital or idiopathic origins [7]. Urethral strictures in women are less frequent compared to men due to their shorter urethral length (∼5 cm in women versus ∼20 cm in men), with a prevalence of 3–8 % overall, 4–13 % in women with bladder outlet obstruction [[8], [9], [10], [11]]. and 0.1–1 % in women with lower urinary tract voiding symptoms [12]. Women with urethral stricture disease often present with a weak stream, dribbling, recurrent urinary tract infections, pain localized to the urethra, urgency, frequency, dysuria, hesitancy, overflow urinary incontinence, urinary retention, and/or elevated postvoid residual [13,14]. Etiologies of urethral strictures in women include iatrogenic causes resulting from urogynecological procedures such as sling procedures, transvaginal urinary fistula repair, diverticulum repair, vaginal deliveries, pelvic radiation, and urethral dilation as well as chronic cystitis and urethritis, idiopathic, trauma, and inflammation [15].

A variety of treatment modalities are utilized for urethral stricture disease with selection criteria primarily dependent on stricture location and length as well as history of prior repairs. Short simple strictures (<1 cm in length) are managed endoscopically, however long (>2 cm in length) complex strictures often require one- or two-stage urethroplasty in both men and women [16]. Endoscopic approaches for urethral stricture repair in both sexes include dilation and cold knife internal urethrotomy to increase urethral caliber [16]. Unfortunately, the success rate of these minimally invasive therapies is low for patients with strictures >2 cm length wherein a 75 % stricture recurrence rate has been reported at 48 months post-op [17]. Short, non-complex urethral defects 1–2 cm in length can be repaired with an end-to-end anastomosis by aligning and joining the normal urethral ends [18]. However, for urethral defects >2 cm in length, onlay or tubularized urethroplasty is often performed with patient-derived, buccal mucosa grafts in which a patch or de novo urinary conduit is fashioned from the donor tissue and surgically integrated into the surrounding host tissue [19,20]. The average stricture length in men has been reported to be 4.8 cm suggesting that the majority of male patients will be candidates for urethroplasty with autologous grafts [21].

Despite the use of buccal mucosa as the gold standard for repair of urethral strictures, this approach is hampered by complication rates as high as 37 % including a recurrence rate of 20.5 % for strictures 4–8 cm in length [22]. In addition, the harvesting of autologous tissues requires secondary surgical procedures, is routinely associated with morbidity at the donor site, and donor tissue volume is limited for reconstructive procedures [23]. Tissue engineered scaffolds derived from decellularized small intestinal submucosa (SIS) and bladder acellular matrices (BAM) have been previously explored as alternatives for urethroplasty in short-term clinical studies [[24], [25], [26], [27], [28]]. These implants, however, have failed to translate into widespread clinical practice due to suboptimal scaffold properties which elicit frequent, deleterious side-effects including fibrosis, graft contracture, and stricture reoccurrence [[24], [25], [26], [27], [28]]. In particular, acellular decellularized matrices are limited in their capacity to promote regeneration of urethral segments >2 cm in length due to poor tissue ingrowth and scar tissue formation [29,30]. Moreover, the success of these biomaterials is restricted to patients with a healthy urethral bed, minimal spongiofibrosis and normal vascularity at the implant site [16]. Unfortunately, these factors are often compromised in patients with long urethral strictures [16]. Given the limitations associated with buccal mucosa grafts and conventional biomaterial configurations, the development of new therapeutic interventions for reconstruction of long urethral strictures is warranted.

A crucial step in vetting prospective biomaterial designs for the repair of long urethral strictures is to assess their ability to promote tissue regeneration and support functional voiding in large animal models which mimic the length and severity of urethral strictures encountered in the clinic. This is important since alterations in the regenerative capacity of host tissues can occur as a function of disease or past injury and can ultimately influence implant functional performance [[31], [32], [33]]. Unfortunately, the vast majority of preclinical studies used to assess biomaterial potential for urethroplasty are in small scale, traumatic urethral defects created in nondiseased, male rabbit models which fail to recreate the fibrotic microenvironment and dimensions of long urethral strictures [16,29]. Moreover, no published preclinical models of female urethral strictures currently exist. Rabbit and dog models of short urethral strictures in males (∼1–2 cm in length) have been produced by open surgery, electroresection using a pediatric resectoscope, and electrocoagulation with or without endoscopy [[33], [34], [35], [36], [37], [38]]. The male rabbit urethra, composed of epithelium and corpus spongiosum, has similar histological morphology and structure to that of humans, however the penile urethra is ∼3 cm long and therefore is anatomically limited in its capacity to create long urethral strictures (4–8 cm) with high recurrence rates encountered in patients [39,40]. Canine models do possess urethras >4 cm [36], and are potential candidates for the creation of a long urethra stricture model, however their status as companion animals raises ethical issues which restricts their widespread use in research settings [41]. Electrocoagulation has been reported to generate short urethral strictures (1–2 cm in length) in male swine amenable to urethroplasty procedures [[42], [43], [44]], however the use of this species to simulate long urethral strictures (≥4 cm in length) in both sexes has yet to be described in the literature. Therefore, the goal of the present study was to develop both male and female porcine models of long urethral strictures via electrocoagulation and characterize outcomes of iatrogenic stricture formation between genders.

## Materials and methods

### Animals and surgical manipulations

Four castrated male (Pigs 1-4 M) and four female (Pigs 1-4F) adult Yucatan mini-swine (∼24 weeks of age, 30–40 kg, PremierBioSource, Ramona, CA) were subjected to vesicostomy creation as well as urethral electrocoagulation injury for formation of long urethral strictures using the methods described below.

### Vesicostomy

Prior to surgery, both male and female animals were fasted overnight and permitted free allowance of water. General anesthesia was initiated by intramuscular injection of 2.2 mg/kg Anased (Lloyd Inc.; IA, United States) and 4.4 mg/kg Telazol (Zoetis Inc.; Parsippany, NJ, United States), and continued by endotracheal 1–4 % isoflurane inhalation. Swine were supine positioned for creation of the vesicostomy opening. The lower abdomen was scrubbed with betadine and 70 % ethanol and covered with a sterile drape. A vertical paramedian incision (3–10 cm in length) was made on the right lower abdominal wall skin and layers were dissected separately to access the abdominal cavity. The bladder dome was grasped with forceps and suspended with stay sutures. A second 5 mm vertical incision positioned 2 cm below the first incision was made and a 22 French Foley catheter was inserted through this orifice into the abdomen. A 1 cm opening was created in the bladder dome and the Foley catheter was introduced into the bladder, followed by filling the balloon with 20 ml saline and subsequently the stoma was closed with 2 purse strings. The bladder was then anchored to the abdominal wall at the vesicostomy site with three 4–0 polyglactin sutures to prevent detachment. Abdominal wall layers and skin incisions were suture closed. The foley catheter was then anchored to the lateral abdominal wall with multiple non-absorbable sutures and fitted with one way check valves (Heimlich, Mila Int. Inc., Florence, KY, United States) to allow for external urine flow.

### Urethral injury

Following vesicostomy creation, electrocoagulation was performed to induce luminal urethral damage and promote stricture formation in both sexes. For male swine, the animals were kept in the supine position and a 1–2 cm vertical skin incision was made below the urethral opening to expose the distal penile shaft. The glans was manually extruded from the foreskin and a 9.5 French rigid cystoscope (Karl Storz 27,030 KB Pediatric Operating Cysto-Urethroscope; Tuttlingen, Germany) was advanced through the urethral meatus. Normal urethral anatomy and length was confirmed using imaging modalities detailed below. Under direct visualization, a 6 cm long and 2–3 mm wide electrocoagulation injury was made from the 3–9 o'clock position ∼2 cm proximal to the external meatus in the anterior urethral spongiosum using a bugbee electrode from the cystoscope. Two small incisions were made on the skin over the penile body and the injury borders were marked with subcutaneous steel rings for longitudinal surveillance of wound healing outcomes. In female swine, animals were maintained in the prone position and the genital confluence was sterilized by betadine application. The length of the urethra was measured using a 5 French ureteral stent and normal anatomy was confirmed prior to injury via cystoscopic evaluations described in following sections. A 23 French urethral resectoscope (AED, Model 8805B-SC, CA, United States) was then introduced into the urethral meatus and electrocoagulation injury ∼5 cm in length in the ventral anterior urethra was performed from the 3–9 o'clock position. The distal urethra located ∼1 cm from the external meatus was kept intact to preserve external urinary sphincter function. Both female and male swine were recovered from anesthesia and maintained on a warming table. Intramuscular Banamine (1.1 mg/kg) was administered post-operatively and a transdermal fentanyl patch (1–4 μg/kg) was applied 24 h prior to the surgery for pain management. In addition, a 3.9 mg/day Oxytrol patch (Merck; Rahway, NJ, United States) was administered to all animals postoperatively to mitigate bladder spasms.

### Retrograde urethrography (RUG) and urethroscopy

Urethroscopic and RUG evaluations were performed prior to surgical manipulations and weekly following electrocoagulation injury to visualize urethral anatomy and the degree of stricture severity. Male and female animals were sedated and anesthesia as well as post-operative analgesics were administered as described above. For male swine, animals were maintained in the supine position, the glans was exposed and a 9.5 rigid cystoscope was inserted through the urethral meatus. Video images were subsequently acquired by an imaging system (Image 1 HUB; Karl Storz, Tuttlingen, Germany) throughout the length of the organ. Following urethroscopy, a 6–8 French silicone catheter was inserted into the external urethral meatus and 1:1 diluted iohexol contrast agent (Omniopaque 300; GE Healthcare, Milwaukee, WI, United States) was instilled. Anterior-posterior retrograde urethrograms were acquired with C-arm fluoroscopy (BV Pulsera; Philips, Eindhoven, Netherlands). For female swine, animals set in the prone position and a speculum was placed into the genital confluence to access the urethral orifice. Urethroscopic surveillance was performed in a similar fashion as described for males. Next, an open ended 14 French catheter was introduced ∼1 cm into the external urethral orifice and the 1:1 diluted contrast agent was instilled, and serial anterior-posterior images were acquired as detailed above. Following confirmation of urethral stricture formation, animals were euthanized and urethral tissues were harvested for histological, immunohistochemical (IHC) and histomorphometric evaluations.

### Histological, IHC, and histomorphometric analyses

Following necropsy, male and female urethras (*N* = 4 per gender) were resected en bloc and divided into proximal, central, and distal segments of equal length dispersed along the axis of the original electrocoagulation injury. Control specimens were isolated from uninjured urethral segments located distally from the site of initial damage. Specimens were then fixed in 10 % neutral-buffered formalin, dehydrated in graded alcohol solutions, and embedded in paraffin. Five micron sections were cut and samples were stained with Masson's trichrome (MTS) using routine histological methods. IHC analyses were performed on parallel sections following antigen retrieval in 10 mM sodium citrate buffer (pH 6.0) and incubation in blocking buffer containing phosphate-buffered saline with 5 % fetal bovine serum, 1 % bovine serum albumin, and 0.3 % Triton X-100 for 1 h at room temperature. Specimens were then stained for 12 h at 4 °C with the following primary antibodies: anti-α-smooth muscle actin (SMA) (1:200 dilution; Sigma-Aldrich, St. Louis, MO), anti-pan-cytokeratin (CK) (1:150 dilution; Dako, Carpinteria, CA), anti-myeloperoxidase (MPO, Abcam, Cambridge, MA, 1:100 dilution), anti-CD68 (Thermo Fisher Scientific, Cambridge, MA, 1:200 dilution), anti-neurofilament 200 (NF200) (Sigma-Aldrich, 1:250 dilution], and anti-CD31 (1:100 dilution; Abcam). For MPO and CD68 detection, specimens were incubated with species-matched, horseradish peroxidase (HRP)-conjugated secondary antibodies and 3,3’Diaminobenzidine (DAB) substrate and then counterstained with hematoxylin. For all other markers, samples were probed with species-matched Alexa Fluor 594-conjugated secondary antibodies (Thermo Fisher Scientific, Waltham, MA) and 4′, 6-diamidino-2-phenyllindole (DAPI) nuclear counterstain. Visualization of stained tissues was carried out with a Zeiss Axio Imager M2 model (Carl Zeiss MicroImaging, Thornwood, NY) and representative fields were acquired with Zen software (version 3.1). Negative controls consisting of parallel tissue specimens incubated with secondary antibodies in the absence of primary antibodies were carried out similarly and produced no significant signal above background.

Histomorphometric evaluations (*N* = 4 per gender and urethral segment) were performed on proximal, central, distal, and control urethral specimens described above using previously published methods [39,40]. Area measurements and image thresholding were carried out on global 5× microscopic fields encompassing the urethral cross-section acquired from 3 serial sectioned specimens per region with ImageJ software (version 1.47). Quantitation of urethral luminal area following electrocoagulation injury was calculated in each damaged segment relative to corresponding control area for each animal following MTS. For IHC analyses, the relative percentages of tissue area stained for markers of interest per total field area was performed in parallel using similar protocols. In addition, the number of NF200+ nerve trunks and CD31+ vessels were calculated across four independent microscopic fields (10×) per urethral sample using comparable methods and normalized to total field area to calculate maker densities.

### Statistical evaluations

Statistical evaluations of quantitative data between groups was performed using the Kruskal Wallis test in combination with the post hoc Dunn's test considering a value of *p* < 0.05 as significant. Quantitative data were reported as mean ± standard deviation (SD).

## Results

**Fig. 1:**
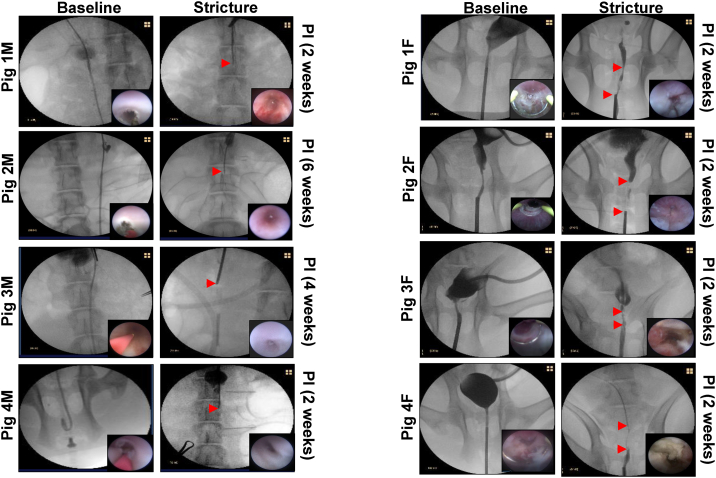
Imaging evaluations of iatrogenic urethral injury and stricture formation. Representative RUG analyses in male [A] and female [B] swine at baseline prior to electrocoagulation (left columns) and at 2–6 weeks post-injury (PI) demonstrating urethral stricture formation (right columns). Arrowheads in both panels demarcate the proximal and distal borders of strictured regions. Insets display endoscopic assessments of injured segments following primary urethral damage (left columns) and at terminal timepoints (right columns).

In both sexes, normal urethral anatomy was confirmed in all animals (*N* = 4 male/female swine) prior to surgery by RUG and urethroscopic analyses (Fig. 1A, B, left columns). Following vesicostomy creation, a focal, partial thickness urethral injury was performed via electrocoagulation over a 6 cm long segment of the male penile urethra. This surgical strategy was utilized since it avoids the tortuosity of the sigmoid flexure present in proximal male urethra thus permitting transurethral instrumentation with a standard urethroscope. Electrocoagulation of female swine urethra was performed in a similar fashion, however due to the shorter length of the female urethra relative to males, the injury region was 5 cm long. All animals survived primary urethral damage and were evaluated weekly for up to 6 weeks by RUG and urethroscopic analyses to determine the kinetics of stricture formation. There were no intraoperative complications noted during vesicostomy and electrocoagulation procedures and all animals were successfully recovered from anesthesia and survived to harvest. External urine flow from suprapubic catheters was apparent in all animals throughout the study period, however catheter reinsertion was necessary in 2 pigs (1F and 2 M) following dislodgement from the vesicostomy.

Post-operative outcomes in swine are summarized in Table 1. All female swine exhibited urinary straining 3–10 days following initial urethral injury which resulted in rectal prolapses <2 cm in length. Prolapses were managed with non-invasive methods including analgesics and constipation mitigation. Urethral stricture formation was detected by imaging modalities in all female swine by 2 weeks following urethral damage (Fig. 1B, right column). In particular, strictures presented as limited extension of contrast agent and/or stenosis along the length of the original electrocoagulation injury following RUG analysis. In addition, Pigs 2F and 3F displayed dilated urethral segments proximal to the injured regions due to putative hydrodistension. Moreover, urethroscopic observations revealed prominent red and edematous areas throughout the injured urethral mucosa in all female swine indicative of chronic inflammatory processes. The mean length of urethral strictures in female swine calculated from RUG photomicrographs was 4 ± 1.4 cm (*N* = 4) with a range from 2 to 5 cm. In males, stricture formation occurred in 100 % of animals (Fig. 1A, right column). However, the onset of urethral stenosis was delayed relative to the female cohort and occurred between 2 and 6 weeks following injury with a mean duration of 3.2 ± 1.8 weeks. In addition, male swine tolerated urethral damage without any clinical presentations of urinary straining or rectal prolapse in contrast to females. Injured urethral mucosa in males also contained red and edematous tissues consistent with ongoing stages of wound healing. RUG and urethroscopic analyses at terminal timepoints revealed all male animals exhibited discrete regions of luminal ablation scattered along the original injury site which impeded extension of contrast agent into the bladder. This scenario precluded quantitation of urethral stricture length from in situ imaging observations since the proximal region of the original injury site could not be penetrated by contrast instillation.

**Table 1:** Post-operative outcomes in male and female swine following urethral injury.

Animals	Study Period	Complications and Management	Imaging Outcomes	Stricture Length
Pig 1F	2 weeks	Dislodgement and reinsertion of vesicostomy catheter at post-op day 11. Rectal prolapse observed at post-op day 13 due to urinary straining.	Mild/Moderate stenosis and inflammed mucosa detected. Limited extension of contrast agent in injured segments.	5 cm
Pig 2F	2 weeks	Rectal prolapse observed at post-op day 12 due to urinary straining.	Mild/Moderate stenosis and inflammed mucosa detected. Limited extension of contrast agent in injured segments.	5 cm
Pig 3F	2 weeks	Rectal prolapse observed at post-op day 13 due to urinary straining.	Mild/Moderate stenosis and inflammed mucosa detected. Limited extension of contrast agent in injured segments.	2 cm
Pig 4F	2 weeks	Rectal prolapse observed at post-op day 3 due to urinary straining.	Mild/Moderate stenosis and inflammed mucosa detected. Limited extension of contrast agent in injured segments.	4 cm
Pig 1 M	2 weeks	None	Complete urethral occlusion and inflammed mucosa observed.	4 cm
Pig 2 M	6 weeks	Dislodgement and reinsertion of vesicostomy catheter at post-op day 18.	Complete urethral occlusion and inflammed mucosa observed.	4 cm
Pig 3 M	6 weeks	None	Complete urethral occlusion and inflammed mucosa observed.	4 cm
Pig 4 M	2 weeks	None	Complete urethral occlusion and inflammed mucosa observed.	4 cm

**Fig. 2:**
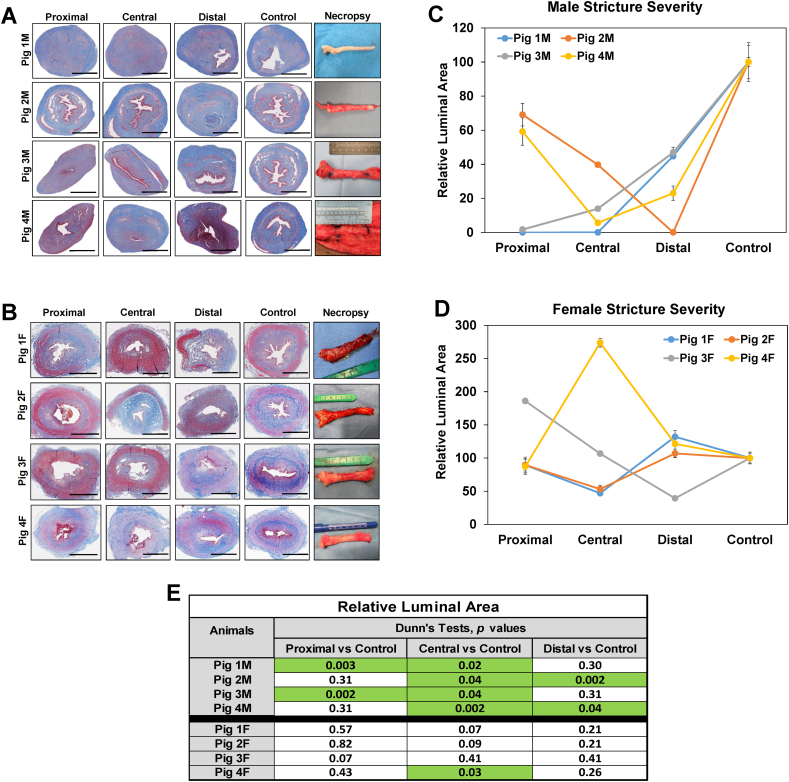
Histological assessments of long urethral stricture formation in male and female swine. [A, B] Gross necropsy specimens and cross-sectional views of MTS-stained, strictured segments (proximal, central, distal) and control regions from male and female urethras. Scale bars = 7 mm for histological panels. [C, D] Quantitation of relative luminal areas in control and injured groups described in panels A and B. Data are presented as means ± standard deviations. [E] Kruskal-Wallis and post hoc Dunn's tests were performed on data described in C. Table displays *p* values from Dunn's analysis with significant regional differences (*p* < 0.05) between strictured regions and respective controls for each animal noted in green highlighted fields. (For interpretation of the references to colour in this figure legend, the reader is referred to the web version of this article.)

Global histological (MTS) evaluations were performed on injured urethral segments as well as control regions to characterize the extent of tissue remodeling and degree of stricture severity (Fig. 2). In contrast to control segments, spongiofibrosis was apparent throughout the proximal, central, and distal areas of injured male urethras with varying degrees of stenosis noted secondary to luminal invasion of collagenous tissues consistent with imaging findings (Fig. 2A). In addition, all male swine demonstrated significant reductions in relative luminal area within central regions of the original injury site relative to controls, whereas 2/5 animals displayed significant attenuation of either proximal (Pigs 1 M, 3 M) or distal (Pig 2 M, 4 M) luminal areas compared to uninjured segments (Fig. 2C, E). Therefore, the mean length of urethral strictures in all males was ∼4 cm based on the length of urethral segments exhibiting significant declines in luminal area relative to controls. In contrast to males, stricture severity was less pronounced in female swine with no significant differences in relative luminal areas noted in injured regions in respect to control segments except for dilation of the central region of Pig 4F (Fig. 2B, D). These data suggest that obstructive uropathy encountered in porcine urethral strictures is primarily due to anatomical obstruction in males, while female pathology is a result of putative functional obstruction.

**Fig. 3:**
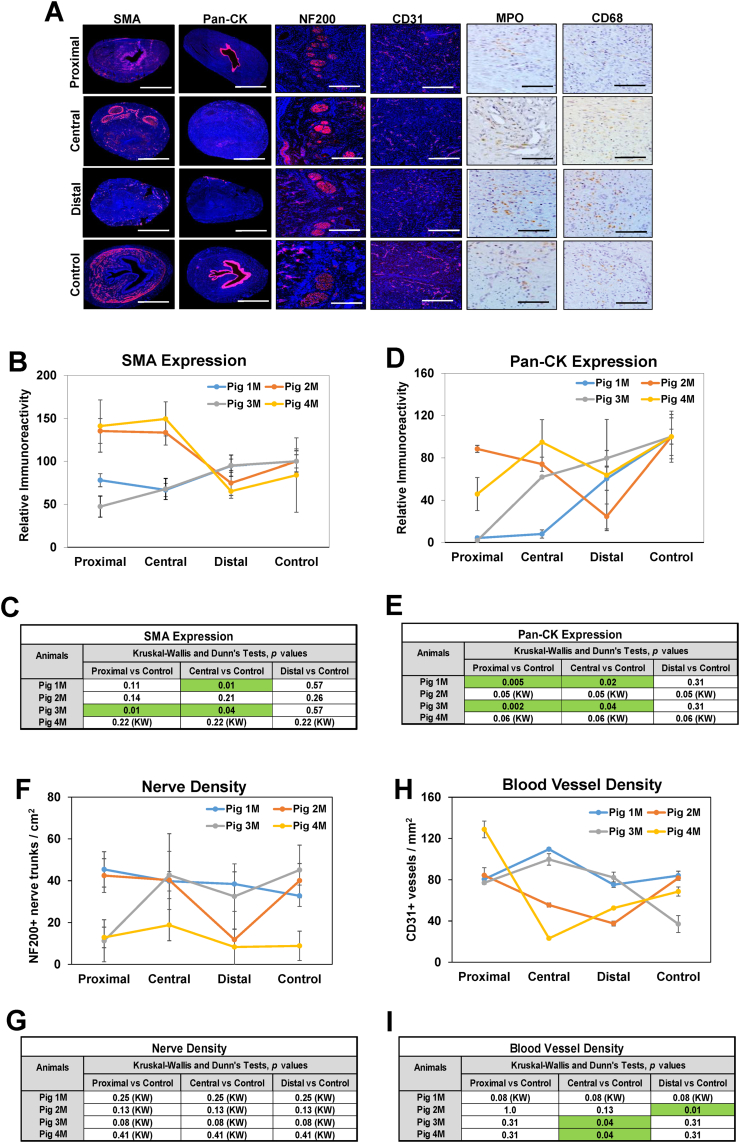
Immunohistochemical and histomorphometric analyses of long urethral strictures in male swine. [A] Representative photomicrographs of selective protein expression in strictured segments (proximal, central, distal) and control regions from Pig 4 M. Markers include smooth muscle contractile protein (SMA), epithelial protein (pan-CK), vascular endothelial CD31 protein, neuronal NF200 protein and neutrophil (MPO) and macrophage (CD68) antigens. For columns 1–4, respective marker expression is labeled in red (Alexa Fluor 594 labeling) with blue representing DAPI nuclear counterstain. For columns 5–6, positive marker labeling is in brown (horseradish peroxidase) with hematoxylin nuclear counterstain in blue. Scale bars for 1st and 2nd columns are 7 mm, 3rd and 4th columns are 600 μm, and 5th and 6th columns are 200 μm. SMA, smooth muscle actin; Pan-CK, pan-cytokeratin; NF200, neurofilament 200; MPO, myeloperoxidase; DAPI, 4′, 6-diamidino-2-phenyllindole. [B, D, F, G] Quantitative assessments of markers displayed in panel A for Pigs 1 M–4 M. Data are presented as means ± standard deviations. [C, E, G, I] Kruskal-Wallis (KW) and post hoc Dunn's tests were performed on data described in panels B, D, F and G. Tables display *p* values from KW or Dunn's analyses with significant regional differences (*p* < 0.05) between strictured regions and respective controls for each animal noted in green highlighted fields. (For interpretation of the references to colour in this figure legend, the reader is referred to the web version of this article.)

**Fig. 4:**
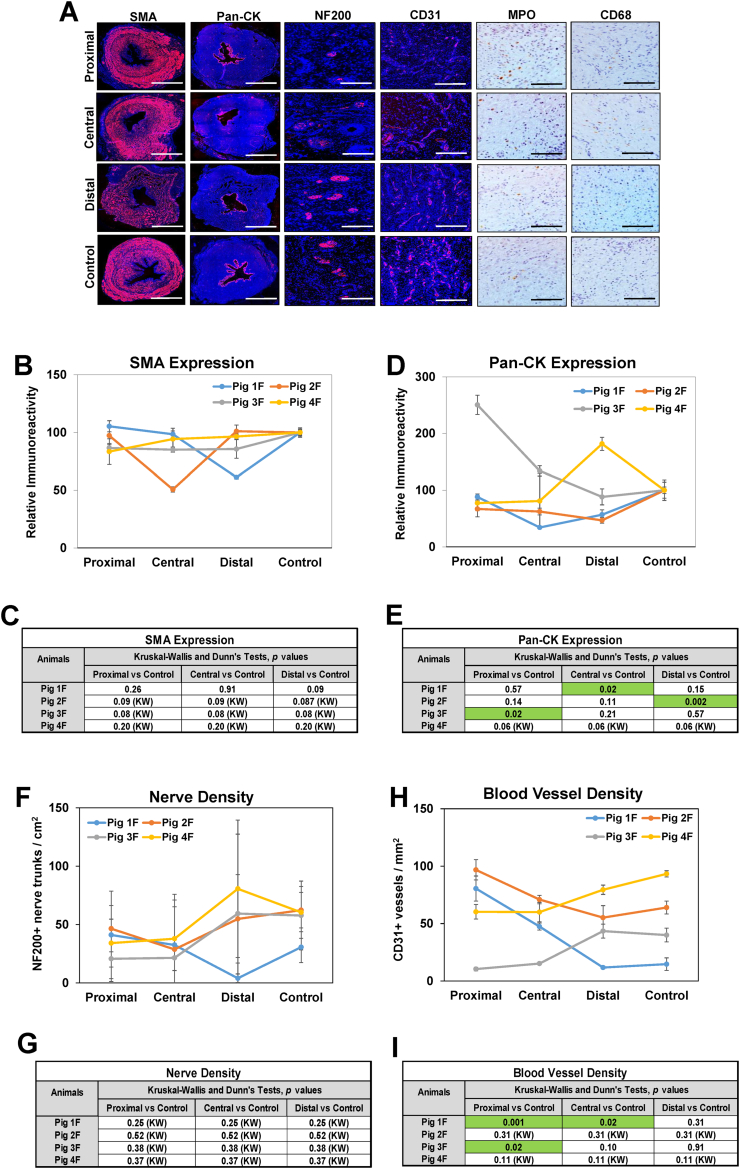
Immunohistochemical and histomorphometric assessments of long urethral strictures in female swine. [A] Representative photomicrographs of selective protein expression in strictured segments (proximal, central, distal) and control regions from Pig 1F. Markers include smooth muscle contractile protein (SMA), epithelial protein (pan-CK), vascular endothelial CD31 protein, neuronal NF200 protein and neutrophil (MPO) and macrophage (CD68) antigens. For columns 1–4, respective marker expression is labeled in red (Alexa Fluor 594 labeling) with blue representing DAPI nuclear counterstain. For columns 5–6, positive marker labeling is in brown (horseradish peroxidase) with hematoxylin nuclear counterstain in blue. Scale bars for 1st and 2nd columns are 7 mm, 3rd and 4th columns are 600 μm, and 5th and 6th columns are 200 μm. SMA, smooth muscle actin; Pan-CK, pan-cytokeratin; NF200, neurofilament 200; MPO, myeloperoxidase; DAPI, 4′, 6-diamidino-2-phenyllindole. [B, D, F, G] Quantitative assessments of markers displayed in panel A for Pigs 1F—4F. Data are presented as means ± standard deviations. [C, E, G, I] Kruskal-Wallis (KW) and post hoc Dunn's tests were performed on data described in panels B, D, F and G. Tables display p values from KW or Dunn's analyses with significant regional differences (p < 0.05) between strictured regions and respective controls for each animal noted in green highlighted fields. (For interpretation of the references to colour in this figure legend, the reader is referred to the web version of this article.)

In both genders, focal regions of epithelial sloughing as well as submucosal fibrosis and infiltration of mononuclear inflammatory cells were observed throughout the walls of damaged urethral sections (Fig. 2A, B). IHC assessments (Figs. 3A, 4A) revealed CD68 + macrophages and MPO+ neutrophils were primarily distributed in the mucosa of injured regions in both male and female urethras. Histomorphometric outcomes of immunostained specimens (Figs. 3B, 4B) demonstrated that 50 % of male and female replicates displayed significant reductions in Pan-CK+ epithelia as well as alterations in vascular density within discrete strictured regions in comparison to relative controls. In addition, proximal and central regions of urethral strictures in 50 % of the male cohort displayed significant declines in SMA + smooth muscle bundles from control levels, whereas no significant changes in smooth muscle content were observed in damaged female urethras. Assessments of nerve densities in experimental groups did not uncover significant alterations between injured and control segments in either sex. These data demonstrate that electrocoagulation injury results in long (∼4 cm in length) urethral stricture formation in both male and female swine, however the extent of pathology severity is dependent on gender, injury location and individual wound healing response.

## Discussion

The aims of this study were to establish male and female porcine models of iatrogenic long (≥4 cm) urethral strictures and determine the impact of gender on wound healing responses to urethral injury. Urinary diversion via vesicostomy was performed in all animals to mitigate deleterious elevation of urinary storage pressures secondary to stricture formation. Longitudinal imaging was conducted to monitor the rate and severity of stricture formation, while histological, IHC and histomorphometric evaluations were carried out to profile gender-specific wound healing patterns. We utilized focal electrocoagulation to induce urethral damage in swine based on past findings in porcine and rabbit models demonstrating this mode of injury was sufficient to generate short urethral strictures (1–2 cm in length) with histopathological features similar to human disease [42]. The length of primary urethral injury was 5 cm in females and 6 cm in males which represented ∼63 % and ∼ 38 % of the total urethral length, respectively based on mean lengths reported for each gender (8 cm in females versus 16 cm in males) [45,46]. Clinical presentations of urethral injury following electrocoagulation were substantially different between male and female cohorts with the latter experiencing severe straining and rectal prolapses. The discrepancy in post-operative outcomes between sexes may be related to the greater proportion of the urethra subjected to injury in females which could have exacerbated urethritis and pain resulting in increased straining during voiding attempts. In addition, putative damage to the pudendal nerve in females during model creation may have also contributed to the higher incidence of rectal prolapse secondary to pelvic floor dysfunction [47].

The mean onset of stricture formation in both sexes (2–3 weeks post-op) was comparable to previous studies in male rabbits and swine which demonstrated the development of short strictures 1–2 weeks post-electrocoagulation injury [33,42]. Despite variations in the size of the original injury site, both genders exhibited urethral strictures with a mean length of ∼4 cm presumably due to axial wound contracture. Similar to human pathology, mucosal damage was evident throughout stricture segments in both cohorts characterized by focal loss of Pan-CK+ epithelia, perturbations in vascular density as well as invasion of CD68 + macrophages and MPO+ neutrophils [42]. However, our results also showed marked variations in the degree of stricture severity between male and female groups. Specifically, male urethras contained stenotic regions with significantly reduced luminal areas compared to controls, while female counterparts showed modest declines in organ caliber and primarily exhibited functional obstruction. Male urethras were also more susceptible to smooth muscle loss following injury than females and displayed qualitatively higher levels of spongiofibrosis in damaged segments. In addition, the smaller caliber of the urethra in males (9.5 French) relative to females (23 French) likely predisposed the former to anatomical obstruction due to aberrant collagen deposition and radial wound contraction in the urethral wall. On the other hand, the presence of filling defects in female strictures may be explained by declines in urethral wall elasticity from poor mucosal healing which could have restricted fluid flow [48,49]. Indeed, functional urethral obstruction has also been observed in hypospadias patients following tubularized incised plate procedure wherein the reconstructed urethral wall exhibits impaired epithelialization and a contracted groove develops which impedes urine transport, but allows for free passage of a rigid catheter [49].

There were a number of limitations in our present study. First, we utilized castrated male swine in our protocols to mitigate aggressive behavior during animal husbandry operations [50]. However, Hofer and colleagues previously showed that testosterone supplementation in castrated rats following urethrotomy led to increased inflammatory responses and myofibroblast proliferation; conditions which may predispose urethras to stricture formation [51]. Therefore, the impact of androgen signaling should be considered in male models of urethral stricture disease to account for the effects of testosterone on urethral healing. Secondly, the use of RUG evaluations alone to quantify stricture length in males was insufficient due to distal luminal obstructions which impeded visualization of the proximal stricture border. Future evaluations of long urethral strictures in swine will also deploy antegrade urethrograms and voiding cystourethrograms to more precisely define stricture dimensions in proximal urethral segments as previously described [52]. Finally, the sample sizes deployed in our investigation were small and primarily focused on histological and imaging assessments from one terminal timepoint. Follow-up mechanistic studies on larger scale cohorts which include early and late stage assessments of stricture formation may shed light on signaling processes that govern pathology development in both genders.

## Conclusions

We have developed and characterized novel male and female models of long urethral strictures (∼4 cm in length) in swine via electrocoagulation injury. These systems mimic the dimensions (> 2 cm in length) of human strictures in need of substitution urethroplasty and display histopathological and imaging features reminiscent of clinical phenotypes [42,53,54]. Our results also uncovered significant variations in the degree of stricture severity between sexes with males exhibiting significant urethral stenosis and smooth muscle loss, while females primarily displayed defects in epithelialization as well as functional urethral obstruction. We anticipate both these preclinical models will provide robust platforms for testing prospective implant designs for reconstruction of long urethral strictures.

## CRediT authorship contribution statement

**Gokhan Gundogdu:** Conceptualization, Methodology, Investigation, Visualization, Writing-original draft preparation, Writing-reviewing and editing. **Travis Nguyen:** Methodology, Formal Analysis, Investigation, Writing-reviewing and editing. **Mando Eijansantos:** Investigation, Writing-reviewing and editing. **Ambika Chaudhuri:** Investigation, Writing-reviewing and editing. **David Barham:** Investigation, Writing-reviewing and editing. **Joel Gelman:** Funding Acquisition, Writing-reviewing and editing. **Joshua Mauney:** Conceptualization, Supervision, Project Administration, Funding Acquisition, Visualization, Writing-original draft preparation, Writing-reviewing and editing.

## Declaration of competing interest

The authors have no financial disclosures or conflict of interests to declare.

## Data Availability

The raw data supporting the conclusions of this article will be made available by the corresponding author upon reasonable request.
